# Molecular Guided Therapy Provides Sustained Clinical Response in Refractory Choroid Plexus Carcinoma

**DOI:** 10.3389/fphar.2017.00652

**Published:** 2017-09-25

**Authors:** Albert Cornelius, Jessica Foley, Jeffrey Bond, Abhinav B. Nagulapally, Julie Steinbrecher, William P. D. Hendricks, Maria Rich, Sangeeta Yendrembam, Genevieve Bergendahl, Jeffrey M. Trent, Giselle S. Sholler

**Affiliations:** Pediatric Oncology Translational Research Program, Helen DeVos Children's Hospital at Spectrum Health Grand Rapids, MI, United States

**Keywords:** choroid plexus carcinoma, molecular guided therapy, mTOR, TP53, IDH2

## Abstract

Choroid plexus carcinomas (CPCs) are rare, aggressive pediatric brain tumors with no established curative therapy for relapsed disease, and poor survival rates. *TP53* Mutation or dysfunction correlates with poor or no survival outcome in CPCs. Here, we report the case of a 4 month-old female who presented with disseminated CPC. After initial response to tumor resection and adjuvant-chemotherapy, the tumor recurred and metastasized with no response to aggressive relapse therapy suggesting genetic predisposition. This patient was then enrolled to a Molecular Guided Therapy Clinical Trial. Genomic profiling of patient tumor and normal sample identified a *TP53* germline mutation with loss of heterozygosity, somatic mutations including *IDH2*, and aberrant activation of biological pathways. The mutations were not targetable for therapy. However, targeting the altered biological pathways (mTOR, PDGFRB, FGF2, HDAC) guided identification of possibly beneficial treatment with a combination of sirolimus, thalidomide, sunitinib, and vorinostat. This therapy led to 92% reduction in tumor size with no serious adverse events, excellent quality of life and long term survival.

## Introduction

In January 2011, a 4 month old female presented with increased emesis and a bulging fontanelle and was diagnosed with Choroid Plexus Carcinoma (CPC) in the right ventricle with metastatic tumor cells present in the cerebral spinal fluid (CSF) and leptomeningeal carcinomatosis. After a complete tumor resection, the patient received the following adjuvant-chemotherapy for 12 cycles (cyclophosphamide, carboplatin, etoposide) according to published CPC therapy and was in remission (Berrak et al., 2011).

Within 1 year of completing chemotherapy the tumor recurred in August 2012 at 23 months of age. MRI revealed new lesions in the right and left ventricles; the spine and CSF were negative for disease. She began treatment with relapse chemotherapy which included bevacizumab, irinotecan, and temozolomide during which time the tumor progressed. This was followed by high dose methotrexate and vincristine which resulted in stable disease but significant vomiting. The patient then received high dose carboplatin and vincristine alternating with ifosfamide and etoposide with stable disease but significant hematologic and infectious toxicity. The patient again received high dose methotrexate and vincristine but at this time had progression of disease and metastases to the spine (Table 1). Notably, the patient had no definitive response to any standard relapse chemotherapy options.

**Table 1:** Relapse chemotherapy treatment strategy, molecular guided therapy strategy, and associated costs.

**Dates**	**Standard treatment**	**Response**	**Adverse events (>Grade 2)**	**Standard drugs**	**Drug cost $**	**Days**	**Additional costs**	**Subtotal $ per month**	**Total $ per month**
Sept–Nov 2012 (3 cycles)	Bevacizumab 10 mg/kg Irinotecan 125 mg/m2 Temodar 140 mg/m^2^ × 5 days	P D	Low blood counts	Temozolamide Irinotecan Bevacizumab Supportive Meds	1,500 75 700 1,500	5 1 1 5	Clinic Facility: $500/day Clinic Exam: $200/day Transfusion: $1,000 each Admission:$4,000/day	Drug $4,823 Medical $5,900	$10,723
Dec–Jan 2013 (2 cycles)	Methotrexate 8 gm/m^2^ Viscristine 1.5 mg/m^2^	S D	Low blood counts Nausea and Vomiting	Methotrexate Vincristine Supportive Meds	1,300 20 1,500	3 1 4		Drug $6,920 Medical $17,600 AE: $8,000	$32,520
Mar–Jul 2013 (2 cycles of each)	Ifosfamide 1,800 mg/m^2^ × 5 days Etoposide 100 mg/m^2^ × 5 days Alternating with: Viscristine 1.5 mg/m2 Carboplatin 560 mg/m^2^	S D	Low blood counts Nausea and Vomiting Infections (Abscess) Anorexia	Ifosfamide Etoposide Supportive Meds Viscristine Carboplatin Supportive Meds	100 20 1,500 20 80 1,500	5 5 5 1 1 3		Drug $4,100 Medical $23,700 AE: $20,000 Drug $4,823 Medical $14,000 AE:$20,000	$47,800 $38,823
Aug–Sept 2013 (1 cycles)	Methotrexate 8 gm/m^2^ Viscristine 1.5 mg/m2	P D	Low blood counts Nausea/ Vomit Anorexia	Methotrexate Vincristine Supportive Meds	1300 20 1,500			Drug $6,920 Medical $17,600 AE: $8,000	$32,520
	**MGT Treatment**			**MGT Drugs**					
Sept 2013–Sept 2016	Thalidomide 4 mg/kg/day Sunitinib 15 mg/m^2^/dose Sirolimus 1 mg/m^2^/day Vorinostat 200 mg/m^2^/dose Supportive Meds	P R	Low blood counts	Thalidomide Sunitinib Sirolimus Vorinostat Supportive Meds	12,566 2,123 1,252 969 0	28 21 28 14	1 Clinic Exam: $220 Sequencing Cost: $2000	$18,910	$19,130

*PD (Progressive Disease), SD (Stable Disease)*.

## Background

Choroid plexus carcinomas (CPCs) are rare, aggressive brain tumors arising from the cerebral ventricular epithelium and comprising 10–20% of intracranial tumors in children less than 1 year of age. The annual incidence is 0.3 cases per million (Sun et al., 2014). CPCs are associated with a poor prognosis with the 5-year event-free survival rate at 10–50% dependent on extent of surgical resection (Wrede et al., 2007; Sun et al., 2014). Adjuvant chemotherapy may be beneficial in CPC (Wrede et al., 2007), but it remains to be determined which agents are the most beneficial. The genetic basis of CPC is poorly understood. CPC is known to have a strong association with Li-Fraumeni Syndrome and *TP53* mutations, but understanding of the underlying biology and molecular alterations in these cancers is incomplete. While genome-wide sequence variation, copy number alteration, or methylation have been reported (Rickert et al., 2002; Ruland et al., 2014; Merino et al., 2015; Tong et al., 2015) we do not yet have a comprehensive description of the genomic landscape of CPC. Approximately 50% of patients have—*TP53* mutations, while CPCs in patients without a mutation in *TP53*, harbored other alterations in the p53 pathway, suggesting that p53 signaling dysfunction is involved in CPC formation (Tabori et al., 2010). Patients with *TP53* mutations had a worse prognosis, with 100% survival in patients with *TP53* wild type tumors and negative TP53 immunostaining, and 0% survival in patients with TP53 *i*mmunopositivity, a marker for TP53 dysfunction (Tabori et al., 2010).

CPCs are aggressive tumors and have a high incidence of recurring and spreading to multiple regions of the body (Ogiwara et al., 2012). Lack of epidemiological data, few reported cases, and controversies surrounding treatment regimens makes it difficult to establish a standardized therapeutic approach in managing CPC. Currently, total tumor resection is the primary goal for treatment. A study by Bettegowda et al. found that 80% of patients who underwent gross total resection remained disease free (Bettegowda et al., 2012). In spite of total resection and adjuvant therapy, the tumor relapsed in our patient suggesting underlying genetic predisposition.

A need exists for improved targeted therapy options for patients with CPC, especially with mutations in the *TP53* pathway, or other rare cancers. In order to understand the underlying genetic mechanisms of a malignancy genomic sequencing and analysis may lead to identification of novel therapies or repurposing of older medications. One such approach is described here with targeting the tumor biology directed therapy rather than the conventional chemotherapy. It is important to report upon these methods and those who have responded when targeted therapy has been used. Here we report one such exceptional responder.

## Discussion

Resection of one of the progressing tumors was performed and this patient was enrolled on Molecular Guided Therapy NMTRC008 study “Feasibility Trial Using Molecular Guided Therapy for the Treatment of Patients with Relapsed and Refractory Childhood Cancer” after obtaining written informed consent for study and written informed consent was obtained from the patient for the publication of this case report (Clinical Trial Indentifier: NCT01802567, Study ID: NMTRC008). This study was conducted under FDA approval for IDE G100111. Patient safety was evaluated by monitoring of adverse events and response was determined by radiological examination with serial MRI of the brain. The tumor was sent for DNA and RNA sequencing and the genomic analysis was discussed in a Molecular Tumor Board where a precision medicine therapy was designed for this patient.

### DNA mutation analysis

Tumor samples from the patient were analyzed for DNA mutation using Ion AmpliSeq™ Cancer Hotspot Panel v2 (Thermo Fisher Scientific, Waltham, MA) on Ion Torrent (Thermo Fisher Scientific) at the Spectrum Health Laboratory. Whole-exome sequencing (WES) was performed at The Translational Genomics Research Institute (TGen) through hybridization using SureSelect Human All Exon 50 Mb kit (Agilent Technologies, Santa Clara, CA) and sequenced on the Illumina HiSeq2000 using paired-end read chemistry and read lengths of at least 105 bp.

**Figure 1:**
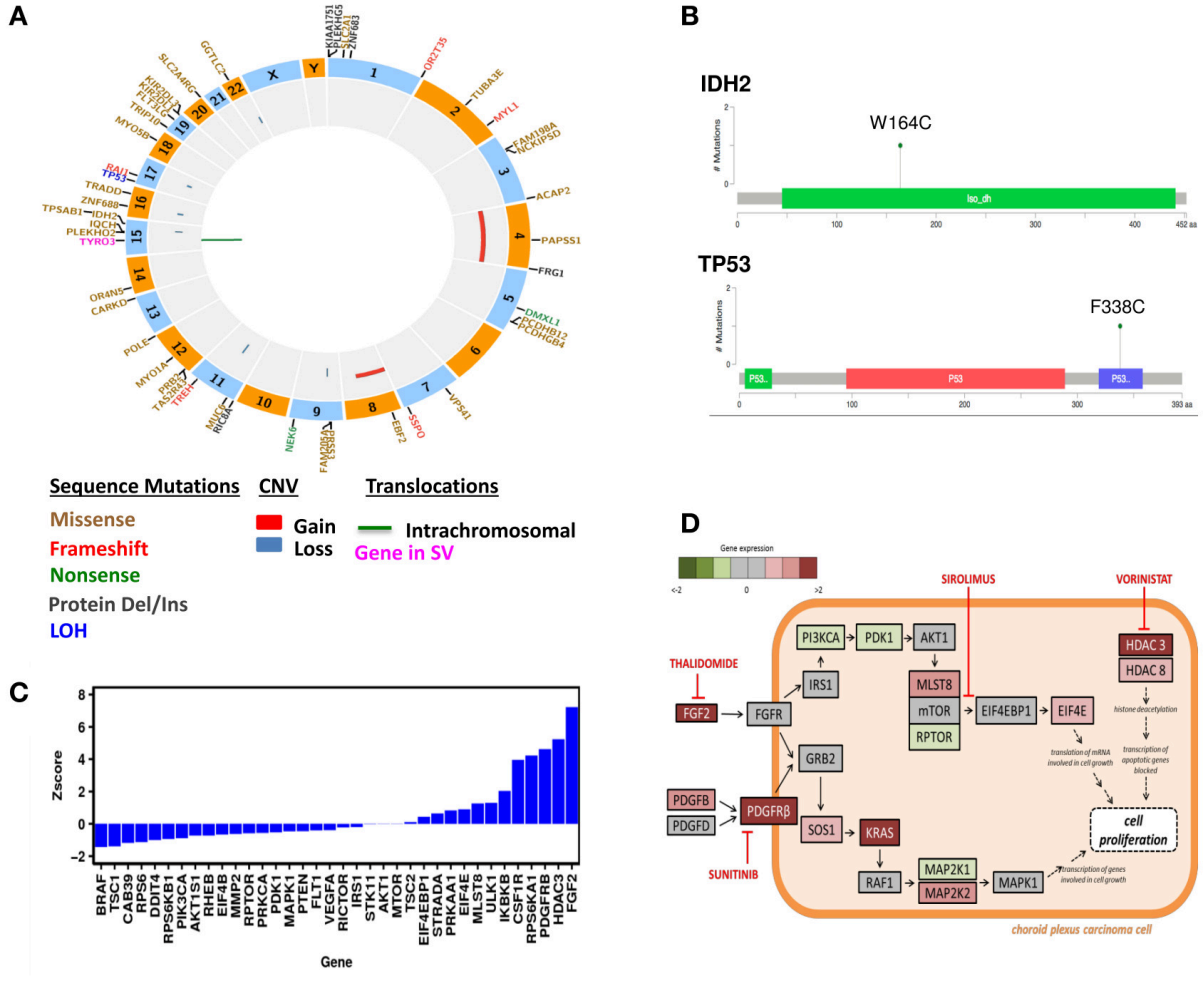
Genomic Analysis of the CPC patient**. (A)** Circos plot of WES results. **(B)** Mutation Diagrams of IDH2 and TP53 (Tetramerization) in context of protein domains. **(C)** Differential gene expression in the CPC patient. Gene expression levels were compared to a normal whole body reference composed of 45 normal tissues and assigned as *Z*-scores. **(D)** Schematic presentation of signaling pathways affected in the CPC patient's tumor and drug targets. These pathways include mTOR, PDGFRB, FGF2, HDAC3, and HDAC8. The treatments chosen for this patient include Sunitinib, Thalidomide, Sirolimus, and Vorinostat.

The overall somatic exome mutation burden was low (Table 1, Figure 1A, and Table S1). We found only eight somatic missense substitutions, three frameshift and four somatic insertions/deletions predicted to alter protein sequence at >20% variant allele frequency (VAF) (Table 1). Somatic alterations between 10–20% VAF (Table S1), Loss of Heterozygosity (Table S2), and Germline (Table S3) are reported as Supplementary. Both sequencing technologies identified amino acid substitution *IDH2*^W164C^ at 23% VAF in the tumor tissue (Table 1, Figure 1B). *IDH2* was recognized as cancer genes in a study involving 21 tumor types (Lawrence et al., 2014).

Exome sequencing of normal tissue found *TP53* amino acid substitution F338C at 51% VAF, demonstrating heterozygosity in the germline. Ion Torrent as well as exome sequencing found this substitution at 81% VAF in the tumor (Table 2, Figure 1B). Exome sequencing of the tumor/normal pair established loss of heterozygosity based on Fisher's exact test (*p* < 0.01), consistent with the results of Ion Torrent sequencing at much higher coverage. This variant has not been observed in large scale population sequencing studies and is predicted to have functional consequences based on PolyPhen2 and SIFT. It seems reasonable to conclude that the combination of (1) a damaging and rare variant in germline with (2) somatic alteration of the wild type allele (loss of heterozygosity) in the tumor played a role in the development of this tumor. Overall, no known targetable mutations were found for both somatic and germline variation associated with cell proliferative disorders.

**Table 2:** Description of Somatic acquired point mutations and rare polymorphism detected in CPC patient by whole-exome sequencing.

**Gene chr:start**	**dbSNP**	**Ref**	**Alt**	**Classification**	**AA change**	**Tumor (normal) read depth**		**Tumor VAF (%)**	**Polyphen2**	**SIFT**	**Mutation taster**
*TREH* 11:1185290 44	rs11448549	C	CG	Frameshift	G569fs	55 (74)		94	–	–	–
*RIC8A* 11:209894	rs3832797	ACCC	A	ProteinDel	P209del	43 (44)		82	–	–	–
*KIAA1751* 1:1900106	rs61233860	T	TCTC	ProteinIns	K404dup	38 (27)		62	–	–	–
*OR2T35* 1:2488019 44	rs143010547	TCAGC ACG	T	Frameshift	C203fs	43 (41)		51	–	–	–
*GGTLC2* 22:229892 70	rs2330126	G	A	Missense	D75N	21 (28)		50	B	T	D
*FAM205A* 9:3472405 9	rs117821239	G	A	Missense	H1060Y	43 (98)		38	P	T	P
*CARKD* 13:1112908 34	–	G	T	Missense	R380L	35 (28)		35	–	–	–
*SSPO* 7:1495185 32	rs11353848	TC	T	Frameshift	Q4202fs	49 (43)		33	–	–	–
*PLEKHG5* 1:6529182	rs113541584	TTCC	T	ProteinDel	E802del	37 (28)		28	–	–	–
*NCKIPSD* 3:4871634 0	–	C	T	Missense	E588K	47 (67)		27	D	D	D
*OR4N5* 14:2061199 4	–	T	G	Missense	F34V	46 (68)		27	D	T	N
*ZNF683* 1:2669128 6	rs372936882	CCCAC CGAGC GCTGG GGTGC CCCAG	C	ProteinDel	L243_ W250d el	52(22)		24	–	–	–
*IDH2* 15:906318 61	–	C	A	Missense	W164C	30 (22)		23	D	D	D
*FAM198A* 3:4307387 3	–	G	A	Missense	A40T	85 (108)		22	B	T	N
*PRB2* 12:1154600 6	–	G	A	Missense	P336S	33 (22)		20	B	–	N
**RARE POLYMORPHISM DETECTED AS LOSS OF HETEROZYGOSITY**											
**Gene chr:start**	**dbSN P**	**Ref**	**Alt**	**Classification**	**AA change**	**Tumor (normal) read depth**	**Normal VAF (%)**	**Tumor VAF (%)**	**Polyphen2**	**SIFT**	**Mutation taster**
*TP53* 17:7574014	–	A	C	Missense	F338C	35 (52)	51	81	D	D	D

*Ref, Reference Allele; Alt, Alternative (Tumor) Allele; AA, Amino Acid; VAF, Variant Allele Frequency; ProteinDel, in-frame deletion; ProteinIns, in-frame insertion; Polyphen2- B, Benign; P, Possibly damaging; D, Probably damaging; SIFT- D, Deleterious; T, Tolerated; MutationTaster- D, Disease_causing; N, Polymorphism; P, Polymorphism_automatic. Alterations with VAF > 20% and decreasing order included*.

### Copy number variation

Several segmental changes consistent with chromosomal instability were identified using WES. This includes large-scale gains in chromosomes 4 (p11-q35.2) and 8 (p11.1-q22.2) as well as focal losses at 9p, 11q, 15q, 16q, 17q, and 22q (Figure 1A, Figure S1). Among the genes localized within large-scale gains are well-established oncogenes: *FGFR1, FGFR3, KIT, PDGFRA*.

### Pathway analysis and drug targets

The resected relapsed tumor sample was sent to the CLIA-certified Clinical Reference Laboratory (CRL) for mRNA expression analysis using U133 2.0 Plus GeneChip (RIN = 9.8). A portion of the same RNA preparation was used for RNA-Seq obtained using Illumina HiSeq2000 at Translational Genomics Research Institute (TGen). The RNA expression levels were compared to a normal whole body reference composed of 45 normal tissues. Differential expression data was interpreted in the context of systems biology annotation for the purpose of identifying activated cellular processes targetable by drugs. Differentially expressed genes are presented as a waterfall graph (Figure 1C). To investigate relationships, mechanisms and functions encoded by differentially expressed genes, *z*-scores from RNA expression were further analyzed by QIAGEN's Ingenuity Pathway Analysis (IPA, http://www.qiagen.com/ingenuity). IPA identified activation of mTOR signaling pathway (*z*-score = 2.7). IPA Causal analytics tools predicted activation of *TP53* (*z*-score = 3.7) as an Upstream Regulator, raising the possibility that the consequences of the TP53 mutation are complex. IPA also predicted activation of *EIF4E* (*z*-score = 3.2) and “inhibition” of sirolimus drug (*z*-score = –4.3) by Causal Network Analysis. Our patient also had overexpression of targetable genes namely *PDGFRB* (*z*-score = 4.6), *FGF2* (*z*-score = 9.8), histone deacetylase *HDAC3* (*z*-score = 5.2), and *HDAC8* (*z*-score = 2.1).

Drug treatments were identified based on gene expression profile of the patient's tumor (Figure 1D). These genome-wide *Z*-scores are used as input to OncInsights drug identification service (Intervention Insight LLC, Waltham, MA). The OncInsight's algorithms are based on biomarker rules, drug target expression, network-based methods, drug response, and drug sensitivity signatures (Saulnier Sholler et al., 2015).

### Molecular tumor board decision

Based on the genomic analysis of the subject's tumor, the molecular tumor board consisting of oncologists, pharmacists, bioinformaticians, and researchers, discussed the patient's previous therapy and current condition. Given the incurable nature of the disease and extensive inpatient therapy the patient previously received which caused significant toxicity, the family requested only oral medications to be prescribed to allow time at home. A treatment regimen was chosen with regards to safety, low toxicity, and targeted mechanism. The chosen pharmacologic agents included sirolimus (targeting mTOR), thalidomide (targeting FGF2), sunitinib (targeting PDGFRB), and vorinostat (targeting HDAC) per the overexpression of pathways and genes shown in Figure 1D.

### Treatment course and adverse events

The therapy was given as described in Table 1 as continuous 28 day cycles. This oral treatment combination was well-tolerated with no serious adverse events or admissions and excellent quality of life. The adverse events noted include expected Grade 3 neutropenia and thrombocytopenia in cycle 3, 10, 15, and 19. In cycle 3, vorinostat was adjusted to 5 days/week and then in cycle 11 the vorinostat was adjusted to 4 days/week to prevent thrombocytopenia. A decision was made in cycle 19 to discontinue vorinostat and at the end of cycle 24 to discontinue sunitinib (due to long term risk of secondary cancers). The patient continues on thalidomide and sirolimus for another 15 cycles without adverse events.

### Radiological response—MRI

**Figure 2:**
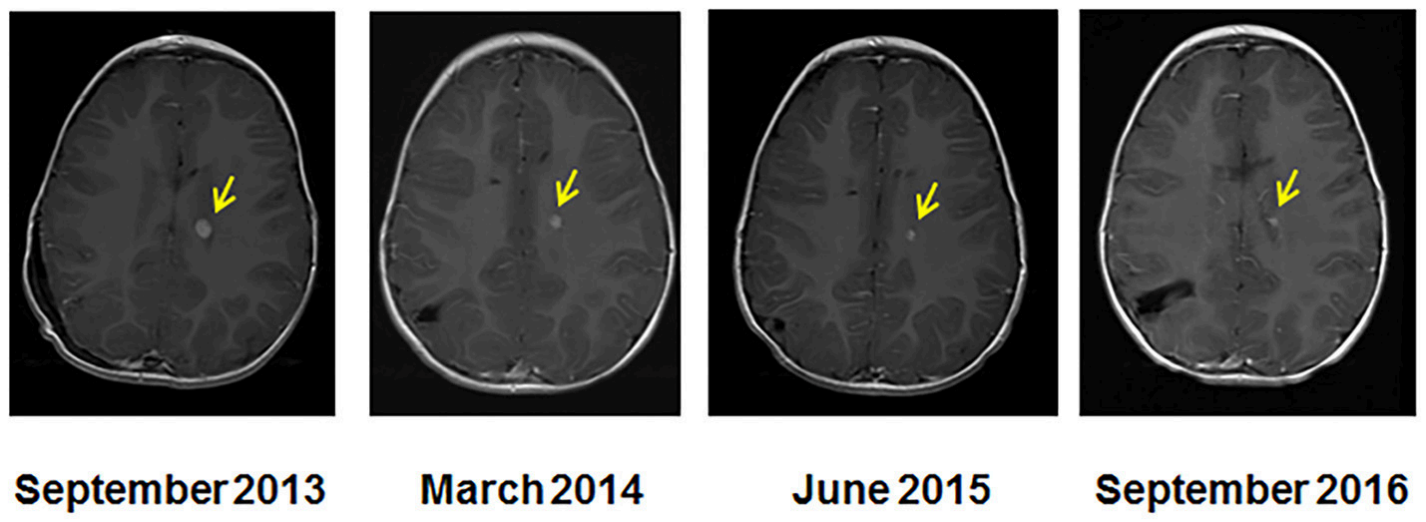
Serial MRI imaging of the CPC patient. MRI at the start of molecular guided therapy regimen (left, Sept 2013), at 7 months (March 2014), at 20 months (June 2015), and at 36 months (September 2016). The arrows indicate the location of the tumor and size reduction over the course of treatment.

After 36 months of the MGT treatment, the patient's MRI showed a 92% tumor reduction from 7.1 × 5.3 × 8.9 mm (335 mm^3^) to 3 × 3 × 3 mm (27 mm^3^) (Figure 2). The spinal tumor and disease noted in the CSF cleared. It is unclear whether the residual mass is active tumor, matured tumor or fibrosis but it is noted that this has not increased in size while off therapy for 1 year without additional treatment.

### Cost of treatment

To assess the overall cost of this repurposed drug therapy we added the drug cost, the cost of inpatient or outpatient care during drug administration, supportive medications and the cost of caring for complications of therapy such as fever and neutropenia, transfusion or continued hospitalization for control of nausea and vomiting. We did not include additional costs such as loss of income to the family from missed days of work, or other incidental costs. We averaged the cost of each therapy per month of treatment and show the results in Table 1. While the MGT required medications of thalidomide, sunitinib, sirolimus, and vorinostat, which were in general more expensive than most chemotherapy medications (except Carboplatin and Avastin), the overall cost of therapy was less each month. One month of MGT cost $19,130 while the high-dose Methotrexate/Vincristine cost $32,520/month, Ifosfamide/Etoposide cost $47,800/month, and Vincristine/Carboplatin cost $38,823/month. Only Temodar/Irinotecan/Avastin was less expensive at $10,723/month.

### Genomic analysis

Genetic basis of CPC is not fully understood, however, it is suggested that germline alterations of *TP53* predispose to CPC in humans (Sevenet et al., 1999; Olivier et al., 2003; Tinat et al., 2009; Custodio et al., 2011). This was further shown in animal models that ablation of *TP53* function causes CPCs in mice (Brinster et al., 1984; Saenz Robles et al., 1994). Copy number alterations that we identified, large scale gains in chromosomes 4 and 8 as well as a focal loss in chromosome 22q, have been described previously in CPC (Rickert et al., 2002; Ruland et al., 2014; Merino et al., 2015; Tong et al., 2015). In evaluating expression data, Koos et al. (2009) demonstrated activation of PDGFRB in CPC by comparison with choroid plexus papilloma, and quantified response to imatinib in cell culture. Indeed, we found a rare germline missense mutation of *TP53*^F338C^ in our patient. This variant has not been reported in large scale population sequencing studies. PolyPhen2 and SIFT analysis predicted this missense variant as a damaging mutation that may have pathological consequences. One report suggested this mutant may retain partial transactivity (Kawaguchi et al., 2005). Our analysis further predicted that the patient's tumor expressed only mutant *TP53* with loss of heterozygosity of the other wild-type *TP53* allele. *TP53* mutations with loss of heterozygosity often have high metastatic and chemotherapy resistant properties due to accumulation of mutant *TP53* with oncogenic gain-of-function (Gonzalez et al., 1995; Alexandrova et al., 2017). A study reported that whereas prognosis was excellent for CPC patients with wild type *TP53*, 5-year overall survival of patients with *TP53* immunopositivity (*TP53* dysfuntion) dropped to 0% (Bettegowda et al., 2012). These findings were consistent with our patient whose tumor relapsed in <2 years and it was refractory to standard aggressive relapse treatments.

We also identified a novel somatic missense mutation on *IDH2*^W164C^. *IDH* mutations have been found in other cancers particularly gliomas and acute myeloid leukemia (Dang et al., 2010; Yang et al., 2012). The association of *IDH* mutations and *TP53* in tumorigenesis has been explored in gliomas. The majority of the literature examined *IDH1*, which occurs more frequently. However, Yan et al. did find that 80% of anaplastic astrocytomas and glioblastomas with a mutation in *IDH1* or *IDH2* also had a mutation in *TP53* (Yan et al., 2009). Though the association of *IDH1* and *TP53* has been examined in the literature, the role of *IDH2* remains more difficult to characterize given the relatively few tumors that are *IDH2* mutated. The role of *IDH2* missense mutation at tryptophan 164 identified in our patient is not known. However, this mutation remains an interesting target to study the tumorigenic mutants of *IDH2*.

Although several compounds have been developed as *TP53* mutant inhibitors (Parrales and Iwakuma, 2015), no specific inhibitor has been identified for the particular *TP53* mutation found in our patient. Similarly, there was no inhibitor for the *IDH2* mutation seen here. This led us to focus our effort toward differentially expressed genes. Pathway analyses identified biological processes and signaling pathways that were significantly enriched for genes of which expressions were altered in the patient's tumor including *mTOR* pathway, *PDGFRB, FGF2*, *HDAC3*, and *HDAC8*. Interestingly, there appears to be a correlation between mutations identified and pathways/ genes altered in the subject's tumor. For example, activation of wild type TP53 was shown to inhibit mTOR activity and its downstream targets (Feng et al., 2005). Constitutive activation of mTOR pathway in our subject's tumor may presumably be attributed to loss of normal TP53 function. A study found that missense mutations in the *TP53* gene caused induction of *PDGFRB* and metastasis (Weissmueller et al., 2014), similar to what we observed in our patient. These findings emphasized that targeting aberrant pathways is a viable option for the treatment of patients.

Anti-cancer activity of Sirolimus and other mTOR inhibitors have been explored in many cancers (Polivka and Janku, 2014) but very little is known in CPC. Our patient's tumor demonstrated overexpression of *EIF4E*, an oncogene and downstream molecule of *mTOR* (Dowling et al., 2010). Targeting *mTOR* with sirolimus was shown to inhibit *EIF4E* (Martin et al., 2014). We assumed this will potentially block cell proliferation. *FGF2*, a known angiogenic factor, was highly upregulated (*z*-score = 9.86) in our patient's tumor, making it an important target. Thalidomide is a potent inhibitor of angiogenesis and *FGF2*. Thalidomide was withdrawn from the market due to teratogenicity but, in recent years, there has been a renewed interest in the use of thalidomide as an antitumor agent. The main use of thalidomide is in the treatment of multiple myeloma (Rajkumar et al., 2006). In pediatric brain tumors, thalidomide given as a metronomic therapy in combination with other agents has shown some response (Peyrl et al., 2012; Porkholm et al., 2014). A recent study demonstrated successful treatment of refractory metastatic gastroesophageal adenocarcinoma with thalidomide in combination with rapamycin (sirolimus). The decision to include sunitinib and vorinostat was based on our findings that *PDGFRB* and *HDAC3/8* were overexpressed in our patient's tumor. Sunitinib is an oral small molecule tyrosine kinase inhibitor that has activity against *PDGFRB*, a proto-oncogene that can be activated in cancer cells (Chow and Eckhardt, 2007). Vorinostat is a known pan *HDAC* inhibitor (Conti et al., 2010). It has been used in several brain tumor clinical trials both as single agent therapy and in combination with other drugs (Bezecny, 2014). Interestingly, *HDAC* inhibition through vorinostat was shown to induce degradation of mutant p53 in cancer cells (Marks, 2007; Li et al., 2011). These results further emphasized that there are overlapping pathways between mutations observed in our patient's tumor and biological pathways altered. Treatment with this combination resulted in a sustained response in our patient with excellent quality of life. These findings suggest that this drug combination is a reasonable adjuvant treatment option for relapsed CPC.

## Concluding remarks

Molecularly targeted therapies tailored to the patient's genetic profile offer a novel approach to obtain improved survival outcomes. In this case study, we report a child with recurrent and metastatic CPC. Due to the refractory and incurable nature of the disease, the patient was enrolled on a clinical trial studying molecular guided therapy. Under this protocol, we molecularly profiled the subject's tumor. Genetic variations as well as a number of genes highly over and under expressed relative to normal tissues were identified, which led to a therapeutic plan. After 36 months of the molecular guided therapy treatment, the patient's MRI showed 92% tumor reduction and the metastatic tumor was cleared. The residual 3 mm nodule remaining is not resectable and may no longer be active tumor as this has not increased in size off therapy. The patient, now 6 years old, continues to thrive 1 year after completion of molecular guided therapy treatment (4 years from enrollment on study).

Using genomic analysis of patient tumors may be one way to identify medications which can be repurposed for new indications. One of the most common criticisms of molecularly guided therapy is the cost of many of the medications, this may not be the case when considering older medications. While some medications may be costlier, our analysis of this patient demonstrated that the overall cost of therapy was less using molecular guided therapy than conventional chemotherapy. When considering the cost of therapy, physicians and payers should consider not only the cost of medications requested, but also the overall cost of therapy including inpatient time and treatment of toxicities.

This case study demonstrates successful treatment of a patient who presented with a refractory and incurable metastatic CPC and highlights the importance of incorporating molecular guided therapy in treatment options for such cases. To the best of our knowledge, this is the first report of adjuvant therapy in this combination with CSF clearing and a sustained response. This clinical report may guide future clinical trials and therapies that are molecularly-guided for each patient.

## Author contributions

Experimental Design of Clinical Trial: GS, JT, JF, AC, and GB. Patient Care of Case: AC, JS, JF, and MR. Bioinformatic Analysis: JB, AN, and WH. Data Analysis: GS, AC, WH, AN, JT, and MR. Writing of Manuscript: AC, GS, JF, MR, and JS. Editing of Manuscript: SY, GS, and GB.

### Conflict of interest statement

The authors declare that the research was conducted in the absence of any commercial or financial relationships that could be construed as a potential conflict of interest.
