# Supplementary material for: Molecular Guided Therapy Provides Sustained Clinical Response in Refractory Choroid Plexus Carcinoma
Source: Front Pharmacol. 2017 Sep 25;8:652. doi: 10.3389/fphar.2017.00652 (PMC5622196; doi:10.3389/fphar.2017.00652)
Supplement: Supplementary file 4 [file DataSheet1.docx]

**Supplementary Legends**

**Fig. S1. Copy number variation (CNV) in the CPC patient tumor.** Realigned BAM files were used to detect CNV. ExomeCNV, a statistical tool that uses coverage and alternative allele frequencies to estimate CNV, was used to detect somatic CNVs from tumor/normal pair. Chromosome regions on x-axis and Log2 ratios on y-axis are represented. Log ratios log(R)> ±0.5 were set for Deletion/Duplication, with region of gain in red and region of loss in green.

**Table S1. Description of Somatic acquired point mutations detected in the CPC patient by whole-exome sequencing.** Alterations with 10-20% VAF are reported here. Abbreviations: Ref, Reference Allele; Alt, Alternative (Tumor) Allele; AA, Amino Acid; VAF, Variant Allele Frequency; ProteinDel, in-frame deletion; ProteinIns, in-frame insertion; Polyphen2, Polymorphism Phenotyping v2; B, Benign; P, Possibly damaging; D, Probably damaging; SIFT, Sorting Intolerant From Tolerant; D, Deleterious, T, Tolerated; D, Disease_causing; N; Polymorphism; P, Polymorphism_automatic.

**Table S2. Description of Loss of Heterozygosity (LOH) detected in the CPC patient.** Alterations are annotated with multiple entries from dbNSFP (<https://www.ncbi.nlm.nih.gov/pubmed/21520341>). Genes are filtered for <5% MAF (1000Gp1, ESP6500, ExAC). Abbreviations: Ref, Reference Allele; Alt, Alternative (Tumor) Allele; AA, Amino Acid; VAF, Variant Allele Frequency; ProteinDel, in-frame deletion; ProteinIns, in-frame insertion; Polyphen2, Polymorphism Phenotyping v2; B, Benign; P, Possibly damaging; D, Probably damaging; SIFT, Sorting Intolerant From Tolerant; D, Deleterious, T, Tolerated; D, Disease_causing; N; Polymorphism; P, Polymorphism_automatic.

**Table S3. Description of Germline mutations detected in the CPC patient by whole-exome sequencing.** Alterations are annotated with multiple entries from dbNSFP (<https://www.ncbi.nlm.nih.gov/pubmed/21520341>). Genes are filtered for <5% MAF (1000Gp1, ESP6500, ExAC). Abbreviations: Ref, Reference Allele; Alt, Alternative (Tumor) Allele; AA, Amino Acid; VAF, Variant Allele Frequency; ProteinDel, in-frame deletion; ProteinIns, in-frame insertion; Polyphen2, Polymorphism Phenotyping v2; B, Benign; P, Possibly damaging; D, Probably damaging; SIFT, Sorting Intolerant From Tolerant; D, Deleterious, T, Tolerated; D, Disease_causing; N; Polymorphism; P, Polymorphism_automatic.
