# Supplementary figures and images for: Molecular Guided Therapy Provides Sustained Clinical Response in Refractory Choroid Plexus Carcinoma
Source: Front Pharmacol. 2017 Sep 25;8:652. doi: 10.3389/fphar.2017.00652 (PMC5622196; doi:10.3389/fphar.2017.00652)

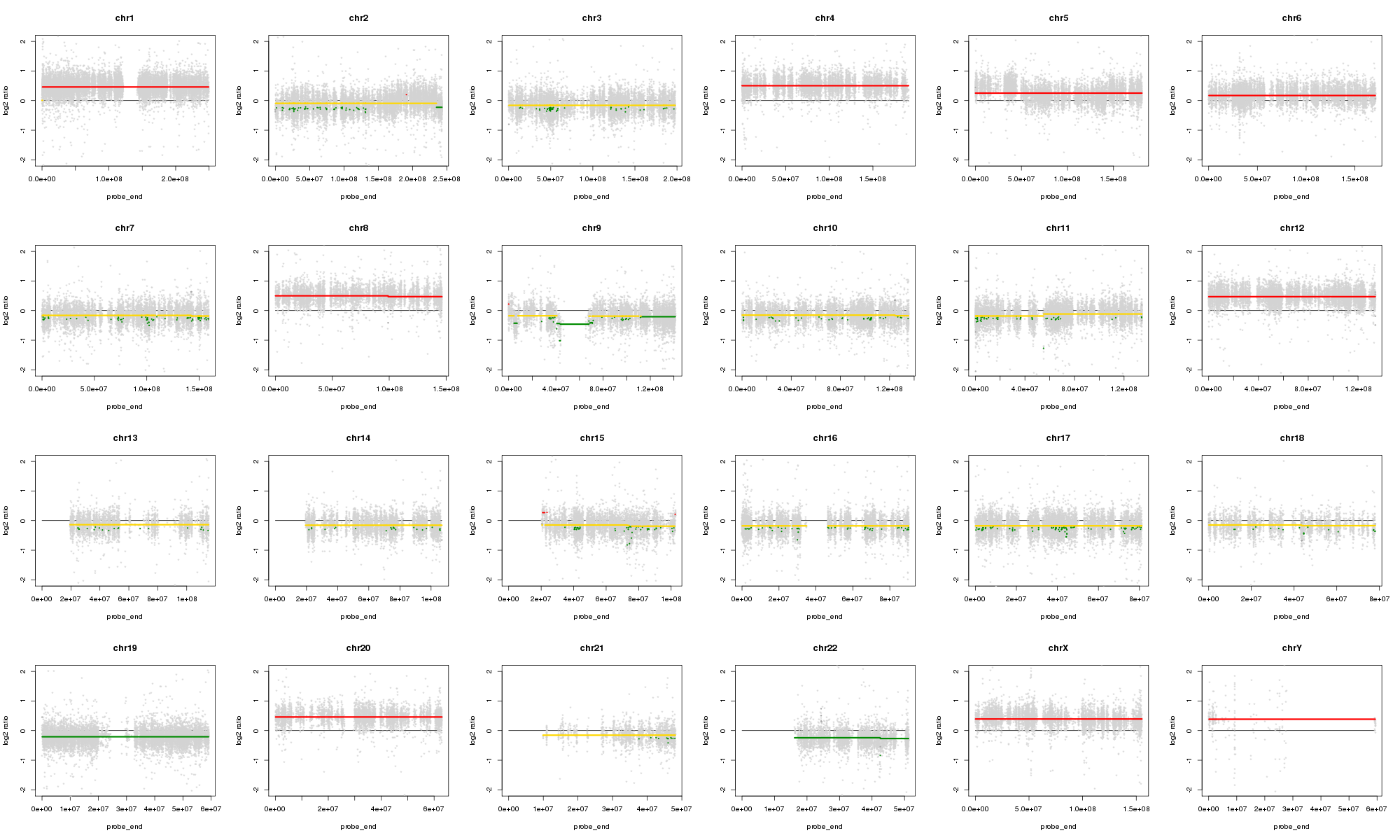

Supplement: Supplementary file 5 [file Image1.png]
